# Supplementary material for: Comparative analysis of the characteristics, care pathways, and outcomes of English and Welsh major trauma patients injured by high versus low energy transfer mechanisms in 2019
Source: PLoS One. 2026 Mar 12;21(3):e0344392. doi: 10.1371/journal.pone.0344392 (PMC12981463; doi:10.1371/journal.pone.0344392)
Supplement: Attachment — Submitted filename: Manuscript Number PONE-D-24-44855R1.pdf [file pone.0344392.s002.pdf]

Overall, I find this an interesting article that draws attention to the changing trauma population. I have a few suggestions for improvement.

- **Titel**: I prefer a shorter title, e.g. Comparative analysis of the characteristics, care pathways, and outcomes of English and Welsh major trauma patients injured by high versus low energy transfer mechanisms
- **Introduction**
- The aim is stated as: "Therefore, the aim of our study was to compare the demographics, care processes and outcomes of patients with severe injuries caused by low and high-energy transfer mechanisms.". I assume that the underlying question is whether the care provided for high-energy trauma is also appropriate or necessary for low-energy trauma cases. I feel this link is missing in the final paragraph of the introduction.
- **Method section**;
    - Please indicate in the methods section which version of the AIS was used.
    - Minor detail: In section Participants: "After study inclusion, a dataset of prospectively recorded variables covering demographics plus injury-related physiological, 7 investigation, treatment and outcome parameters are collated" has to be is collated.
    - **Variables**:
      1. Pre-existing medical conditions do not fall under the category of demographic data, add a category clinical data or change demographics to patient characteristics.
      3. Change penetrating in Penetrating or blunt

- **Results**: The p-values are described, but not showed in the tables. Please add a column in the tables with p-values.

- **Statistical methods:**
    - Under ordinal variables AIS is mentioned. I assume that it concerns the AIS severity, otherwise it is not ordinal.
    - Glasgow Coma Scale can be abbreviated to GCS in the second paragraph.
    - I wonder why the authors did not conduct a multivariable analysis to compare the two groups on outcome and adjust for characteristics of patients and injury (age, comorbidity, severity of injury etc)? It is currently difficult to draw firm conclusions about whether low-fall patients are inappropriately receiving higher-level care. It appears that they are less severely injured but older and therefore more likely to be transported to a non-major trauma center (nMTC). It is in line with the comment of reviewer 1 that it is no surprise that the low fall group has a higher mortality. I would strongly advise to do a more sophisticated comparison between high and low falls to add more depth to the manuscript.
