# Supplementary material for: Comparative analysis of the characteristics, care pathways, and outcomes of English and Welsh major trauma patients injured by high versus low energy transfer mechanisms in 2019
Source: PLoS One. 2026 Mar 12;21(3):e0344392. doi: 10.1371/journal.pone.0344392 (PMC12981463; doi:10.1371/journal.pone.0344392)
Supplement: Attachment — Submitted filename: PLOS One TShanahan Response to Reviewers 24 June 2025.docx [file pone.0344392.s001.docx]

a) If there are ethical or legal restrictions on sharing a de-identified data set, please explain them in detail (e.g., data contain potentially identifying or sensitive patient information, data are owned by a third-party organization, etc.) and who has imposed them (e.g., a Research Ethics Committee or Institutional Review Board, etc.). Please also provide contact information for a data access committee, ethics committee, or other institutional body to which data requests may be sent.

Response:

Data cannot be shared as both: (i) the Data Sharing Agreements established between the TARN (now NMTR) and member NHS Trusts, and (ii) the Section 251 HRA approval for analysis of anonymized TARN data specify the need for data access agreements with third parties. Proposals to access the study data, data dictionary, analytic code, and analysis scripts may be submitted to: [queries@hra.nhs.uk](mailto:queries@hra.nhs.uk). Proposals are subject to review by the TARN Research committee. A Data Transfer Agreement is required, and all access must comply with TARN HRA approval.

| **Reviewer comment** | **Authors response** |
| --- | --- |
| **Reviewer 1** | |
| (i)You show that patients injured by low-energy falls are older, have more TBI and higher mortality, but overall receive "lower intensity" care. Do you think this is adequate, or should it change? | Thank you for the question. If greater intensity means more specialist surgery, particularly neurosurgery then it is uncertain whether the current level is adequate as low energy major trauma patients cared for initially in a trauma unit do not appear to be disadvantaged.  We comment on this in the discussion under “comparisons to existing literature”:  “Other studies on TARN patients injured in low falls (2015-2019) suggest that initial ambulance transport to the nearest hospital emergency department rather than a more distance specialist major trauma centre does not adversely impact outcomes providing later selective secondary transfer is available (25). Furthermore, in older adults with ISS>15 - most of whom are injured in low-energy falls - a frailty score and comprehensive geriatric assessment are more important components of care than the immediate availability of surgical specialists (26,27).”  And in “interpretation of the study and implications”  “The lower rates of MTC admission at any time, surgical intervention, and admission to critical care in the low-energy cohort are difficult to interpret. There may be systematic access barriers to higher level care in older patients. However, it seems that these patients have a much lower requirement for the specialist surgical intervention that is provided in MTCs, as most have head injuries that do not receive surgical intervention (9). This might be due to smaller intracranial haematomas requiring evacuation (2). Moreover, it has already been demonstrated that the ISS had only a low-to-moderate correlation with the need for life-saving interventions (28).”  “The low-energy transfer trauma group may therefore need much more emphasis on multidisciplinary geriatric assessment, care and rehabilitation (26, 27).”  And in “future research”  “Focussing research on early identification of low-energy falls patients that benefit from specialist trauma and neurosurgical input, optimal TBI and chest wall injury management, role of early rehabilitation and better definition of what outcomes are important to patients.” |
| (ii) The death rate in the UK population among 45-49 year olds is something like 2-3 per 1000 population per year, whereas it is 50-70 per 1000 population per year for 80-84 year olds. So maybe the mortality difference is not very surprising? | Thank you for the comment. We have edited the manuscript to highlight mortality is unadjusted. While higher baseline mortality in older adults is expected, our study specifically examines outcomes following severe traumatic injuries (ISS>15), not all-cause mortality in the general population.  We agree that the observed difference in mortality for an older population is not surprising - age is an independent predictor of hospital mortality in trauma survival prediction models (12), however the less intense care received by the energy transfer cohort with higher mortality is notable. |
| (iii) You state that it is challenging triage older patients, but do you think they should be triaged differently, and if so, why? | Thank you this is also uncertain as per our response to your first comment. The systematic review we reference (24) found most tools are not specific or sensitive enough in this area. It is unclear that continuing to focus on prehospital triage of older trauma patients is going to be the best use of resources.  In discussion we further comment:  “This means that the most appropriate treatment location in future should be driven by the availability of these services rather than trauma surgery. In some places multi-disciplinary geriatric trauma care expertise will be concentrated in MTCs, but in some systems local hospitals will have all the skills required, so MTC transfer will not be required. This multi-disciplinary care and rehabilitation has already been shown to improve outcomes after hip fracture (29) so the same model could be extended to treat other low-energy transfer patients.” |
| (iv) You acknowledge that some of your findings are difficult to interpret, but I think you can do more to connect your suggestions for future research with your findings. For example, what type of brain injuries do the low-energy falls patients have and can the differences in neurosurgical intervention be justified? | Thank you in the discussion we have added:  “The findings of CENTER TBI suggest that acute sub-dural haematoma (ASDH) is the predominant CT finding in the low energy cohort with head injury (49% of the low energy TBI cohort with injuries on CT brain -18% described as “large”) reporting that most high and low energy patients with large ASDH received a craniotomy (2). However, CENTER TBI was conducted solely in specialist neuroscience centres - and did not include patients from referring hospitals who were not accepted for transfer. Our “whole system” findings suggest that most low energy major trauma patients with positive CT brain scans after head injury are not accepted for neuroscience/ MTC secondary transfer”  In the conclusion we have added:  “The aging major trauma population, predominantly injured through low-energy falls and suffering severe head injuries, challenges current trauma system design and resource allocation. Although these patients are less likely to require intensive care and surgical interventions, their increasing numbers necessitate an evolution in trauma care pathways. Future research should define which TBI subtypes in low energy falls benefit from neurosurgical intervention; develop patient-centred outcome measures for low-energy falls patients that integrate functional recovery and quality of life and; test care pathways that combine early geriatric assessment with targeted interventions for chest injuries and neurosurgical consultation for high-risk sub-groups.”  In our earlier responses (i)-(iii) we reflect on the appropriateness of this non interventional approach. |
| (v) You write "However, recent studies in high income countries have identified injuries from low-energy transfer mechanisms, such as falls less than two metres, as the most common cause of major trauma presenting to hospital.", but your references are only from England and Wales. Do you have any references from other countries? | Thank you for the comment.  We have made the following change:  “However, recent studies in high income countries have identified injuries from low-energy transfer mechanisms, such as falls less than two metres, as an increasingly common cause of major trauma presenting to hospital (2-5).  We have added the following references:  2) Lecky FE, Otesile O, Marincowitz C, Majdan M, Nieboer D, Lingsma HF, et al. The burden of traumatic brain injury from low-energy falls among patients from 18 countries in the CENTER-TBI Registry: A comparative cohort study. PLOS Med. 2021 Sep 14;18(9):e1003761.  3) Cameron PA, et al. Over view of major traumatic injury in Australia – implications for trauma system design. Injury. 2020 Jan; 51(1):114-121  4) Farrow L, et al. Epidemiology of major trauma in older adults within Scotland: A national perspective from the Scottish Trauma Audit Group (STAG). Injury. 2023 Dec;54(12)  5) Spering C, Lefering R, Bouillon B, Lehmann W, von Eckardstein K, Dresing K, et al. It is time for a change in the management of elderly severely injured patients! An analysis of 126,015 patients from the TraumaRegister DGU®. Eur J Trauma Emerg Surg Off Publ Eur Trauma Soc. 2020 Jun;46(3):487–97.  As a result of these additional references, we have re-numbered the references in the manuscript. |
| (vi) Add spaces between sentences and citations, replace for example "Classifying injuries by energy transfer has received growing attention recently by trauma systems(1–3)" with "Classifying injuries by energy transfer has received growing attention recently by trauma systems (1–3)." | Thanks, we have made those changes throughout the paper as suggested. |
| (vii) Move the TARN eligibility criteria to the Participants section. | Thanks, we have moved it as suggested. See it on page 7. |
| (viii) Please add a Variables section and specify your demographics, care processes and outcomes. From the variables you list under Statistical methods, it looks like you're studying variables that cannot be classified as demographics, care processes or outcomes, for example GCS and ISS. | Thanks for the comment. We have adjusted the title to better reflect the variables. We have also added a variables section as per the suggestion. See page 7:  **Variables**  The following variables were extracted from the TARN database for analysis:   1) Demographics: gender; age; pre-existing medical conditions. 2) Vital signs on arrival into ED: heart rate, systolic blood pressure; GCS; oxygen saturation. 3) Injuries: penetrating; ISS; injury regions with AIS 3+ 4) Pathway: defined as the level of trauma hospital involvement: first MTC hospital; MTC any time to include transfers from other hospitals into an MTC. 5) Interventions: prehospital doctor; seen by a consultant in ED; TXA given; TXA given for those given blood; intubation; time to CT; had operation within 24 hours; time to surgery; critical care admission 6) Patient outcomes: Acute care length of stay (LOS); intensive care unit (ICU) LOS; mortality at 30 days post admission or discharge whichever is earlier |
| (ix) Please add a Data sources section and clarify how the variables were measured. For example, "Pre-existing medical conditions", how were they measured? | Thanks for your comment. They are all extracted from the clinical record (demographics, vital signs and outcomes) only AIS and ISS are derived and PMC using the modified Charlson Co morbidity index (Bouarmra et al EMJ 2015, reference 16), See page 7. |
| (x) Clarify how missing data was handled. | All data were 100% complete other than the Charlson co-morbidity index which was missing in 2% of patients and the missing GCS imputed as per below  Page 8: “For patients with missing GCS an imputation technique based on chained equations and Rubin’s rules was used with the assumption that the mechanism of missingness is at random (MAR) that is the missing value depends on the measured variables. The imputation contained all the variables included in the regression model and the outcome (12).” |
| (xi) Clarify how you dealt with different follow-up times. | TARN follows patients up to discharge from final acute care hospital. All survivors in acute care hospital at 30 days were called survivors even if subsequently died before acute care discharge (as previously published). As per above we have now clarified this in the variables section  “Acute care length of stay (LOS); intensive care unit (ICU) LOS; mortality at 30 days post admission or discharge whichever is earlier” |
| (xii) Add the total number of patients included in the study to the beginning of the Results section. | Thanks. We had added the following: “The study included 30,582 patients.” See page 8. |
| (xiii) Present the amount of missing data for each variable in the Results section. I suggest you include this information in table 1. | Thanks for the comment. In the results on page 9 we address this as follows: “All data were 100% complete other than the Charleson co-morbidity index, which was missing in 2% of patients and GCS, which was missing in 8.2% of patients.” Missing GCS was imputed this is described in methods we have added a footnote to table 1 re missing co-morbidity data. |
| (xiv) Please be consistent in how you present confidence intervals and p-values. For example, in one place you write "Patients injured by low-energy falls (53.6%, n=16,087) were older (median age 80 vs. 47 years", but in another place you write "the low-energy falls cohort were less than half as likely to present with severe impairment of consciousness (GCS 3-8, 6.0% vs. 15.3%; p<0.001)". I suggest you include the p-values in all comparisons, or preferably the differences and associated confidence intervals, especially as you write that because of the large numbers more or less all differences are statistically significant. | Thank you for your comment. We have added in p values in all comparisons. |
| (xv) Spell out MTC the first time it is used. | Thanks, we have done this. |
| (xvi) I don't see that you include anything on the differences in the anatomical distribution of injuries between the two groups in the Results section. As far as I can see, it looks like the low-energy falls cohort has more isolated head injuries whereas the high-energy falls cohort has more multiple injuries. I suggest you include something on this in the text. | Thanks for the comment. On page 8 the first paragraph of the results highlights what you have mentioned: “Patients injured by low-energy falls (53.6%, n=16,087) were older (median age 80 vs. 47 years), less likely to be male (53.8% vs. 73.8%), more likely to have pre-injury co-morbidity (90.4% vs. 56.2**%), less likely to have polytrauma (10.8% vs. 38.2%) and more likely to have a traumatic brain injury visible on CT scan (74.0% vs. 49.8%)** than patients injured by high-energy transfer (46.4%, n=14,495) (Table 1).” |
| (xvii) The paragraph "The low-energy falls cohort stayed on average one day (median nine vs. eight days) longer in hospital but had shorter critical care stays (median three vs. four days). Hospital mortality was 3.5% greater in the low-energy falls cohort (16.1% [95%CI 15% - 17%] vs. 12.6% [95%CI 12% - 13%]; p=<0.0001) (Table 2)" does not match the table, or did you by mistake use the wrong column headings? Because the columns in the table are reversed compared to the paragraph and compared to table 1. | Thank you for spotting this. As suspected, this was a transcription error, and we had the column headings the wrong way round. This has been changed. |
| **Reviewer 2** | |
| Really clear message. Most trauma is low velocity. the ageing population mean its growing. Reference to the NHFD work on improving outcomes maybe needed for all frailty fractures may add to the discussion. | Thanks for the overall feedback. In the discussion page 10 paragraph 5 we have the following line which we think addresses your point: “This multi-disciplinary care and rehabilitation has already been shown to improve outcomes after hip fracture (25) so the same model could be extended to treat other low-energy transfer patients.” |
