# Supplementary material for: Comparative analysis of the characteristics, care pathways, and outcomes of English and Welsh major trauma patients injured by high versus low energy transfer mechanisms in 2019
Source: PLoS One. 2026 Mar 12;21(3):e0344392. doi: 10.1371/journal.pone.0344392 (PMC12981463; doi:10.1371/journal.pone.0344392)
Supplement: Attachment — Submitted filename: Response to reviewers PLoS One TARN manuscript 24 Jan 26.docx [file pone.0344392.s003.docx]

- A rebuttal letter that responds to each point raised by the academic editor and reviewer(s). You should upload this letter as a separate file labeled 'Response to Reviewers'.
- A marked-up copy of your manuscript that highlights changes made to the original version. You should upload this as a separate file labeled 'Revised Manuscript with Track Changes'.
- An unmarked version of your revised paper without tracked changes. You should upload this as a separate file labeled 'Manuscript'.

| **Reviewer comment** | **Authors response** |
| --- | --- |
| **Reviewer 1** | |
| 1. Characteristics actually works quite well in the title, rather than the long "demographics, vital signs, injury patterns" that you have added. My main point in the previous round was that you study many variables which are not demographics, but these are well captured by "characteristics". I would probably keep the title as "Comparative analysis of the characteristics, care pathways, interventions and outcomes of English and Welsh major trauma patients injured by high versus low energy transfer mechanisms in 2019." | Thanks for your comment. We have changed the title as follows:  “Comparative analysis of the characteristics, care pathways, and outcomes of English and Welsh major trauma patients injured by high versus low energy transfer mechanisms in 2019.” |
| 2. Align the aim with the above comment, it still reads as "demographics, care processes and outcomes" and still doesn't cover the vital signs and injury patterns, for example. | Thanks for your comment. We have removed demographics and replaced it with characteristics as per the title.  “Therefore, the aim of our study was to compare the **characteristics**, care processes and outcomes of patients with severe injuries caused by low and high-energy transfer mechanisms.” |
| 3. Include p-values in the abstract. | Thanks for your comment. We have included the p-values and 95% CI for in-hospital mortality.  “**Results:** In 2019, 53.6% (n=16,087) of major trauma patients were injured by low-energy falls. When compared to the high-energy cohort, these affected older patients (median age 80 vs. 47 years; p<0.001), with a higher prevalence of pre-existing comorbidities (90.4% [95%CI 89.9-90.8] vs. 56.2% [95%CI 55.4-57.0]; p<0.001) and traumatic brain injuries (74.0% [95%CI 73.3-74.7] vs. 49.8% [95%CI 48.9-50.6]; p<0.001). Low-energy fall patients were more likely to be initially treated in Trauma Units rather than Major Trauma Centres and received fewer interventions such as surgery and critical care admission. Low-energy falls patients had a higher in-hospital mortality rate (14.0% [95%CI 13.5% - 14.6%] vs. 10.3% [95%CI 9.8% - 10.8%]; p<0.0001). |
| 4. I've put No in response to the question "Has the statistical analysis been performed appropriately and rigorously?". The reason is that you still do not present differences with appropriate uncertainty (for example 95% CIs), but only p-values, for example (58.3% vs. 33.0%; p<0.001). It would be more informative to see an estimate of the difference with uncertainty. | We have added 95%CI for all data in tables 1 and 2 and in the manuscript. |
| 5. Move the information about the missing data to the beginning of the results section, see STROBE. | Thanks for your comment. We have modified as suggested.  “The study included 30,582 patients. All data was 100% complete other than the Charleson co-morbidity index, which was missing in 2% of patients and GCS, which was missing in 8.2% of patients prior to imputation. “ |
| **Reviewer 3** | |
| Title: I prefer a shorter title, e.g. Comparative analysis of the characteristics, care pathways, and outcomes of English and Welsh major trauma patients injured by high versus low energy transfer mechanisms | Thanks for your comment. We have modified the title as per both review comments as follows:  “Comparative analysis of the characteristics, care pathways, and outcomes of English and Welsh major trauma patients injured by high versus low energy transfer mechanisms in 2019.” |
| The aim is stated as: “Therefore, the aim of our study was to compare the demographics, care processes and outcomes of patients with severe injuries caused by low and high-energy transfer mechanisms.”. I assume that the underlying question is whether the care provided for high-energy trauma is also appropriate or necessary for low-energy trauma cases. I feel this link is missing in the final paragraph of the introduction. | Thanks for your comment. We have modified as follows:  “Therefore, the aim of our study was to compare the characteristics, care processes and outcomes of patients with severe injuries caused by low and high-energy transfer mechanisms to determine whether the current model of care is appropriate.” |
| Please indicate in the methods section which version of the AIS was used. | We now indicate that “the 2008 revision of the 2005 AIS dictionary” was used in method |
| Minor detail: In section Participants: “After study inclusion, a dataset of prospectively recorded variables covering demographics plus injury-related physiological, 7 investigation, treatment and outcome parameters are collated” has to be is collated. | Thanks, we have changed are to is. |
| Pre-existing medical conditions do not fall under the category of demographic data, add a category clinical data or change demographics to patient characteristics. | Thanks for your comment. We have changed to characteristics throughout. |
| Change penetrating in Penetrating or blunt | Thanks, we have changed accordingly. |
| Results: The p-values are described but not showed in the tables. Please add a column in the tables with p-values. | Thanks for your comment. We had put a * to explain the p-values in the submitted manuscript. But have now added a column to the table as suggested with p-values. |
| Under ordinal variables AIS is mentioned. I assume that it concerns the AIS severity, otherwise it is not ordinal. | Yes, AIS greater than 3 was used and reported in table 1. |
| Glasgow Coma Scale can be abbreviated to GCS in the second paragraph. | Thanks, we have changed accordingly. |
| I wonder why the authors did not conduct a multivariable analysis to compare the two groups on outcome and adjust for characteristics of patients and injury (age, comorbidity, severity of injury etc)? It is currently difficult to draw firm conclusions about whether low-fall patients are inappropriately receiving higher-level care. It appears that they are less severely injured but older and therefore more likely to be transported to a non-major trauma centre (nMTC). It is in line with the comment of reviewer 1 that it is no surprise that the low fall group has a higher mortality. I would strongly advise to do a more sophisticated comparison between high and low falls to add more depth to the manuscript. | Thank you, the main focus of the paper is a direct comparison of the characteristics, care pathways and outcomes. We considered conducting such a multivariable analysis but have not done so as previous research has shown that once the relevant patient and injury confounders are adjusted for the higher mortality in the low energy cohort is no longer a significant association. We now allude to this in “discussion – comparison to existing literature”  Our analysis confirms the large burden of major trauma from low-energy falls and the predominance of severe head injuries, particularly in patients over 64 years of age, found by this group in England and Wales (9, 17). These findings align with previous work showing that TBI is the most common injury in older trauma patients (9). The results are consistent with a previous study (2), which found differences in care pathways for TBI patients injured by low and high-energy mechanisms and argued for a different approach to improve prevention and care for TBI patients injured through falls less than two metres (2). In this study low energy falls did not independently predict ward or ICU or TBI mortality once the confounders of age, ISS, GCS, pupillary responses, co-morbidity and anticoagulant status were adjusted for in the multivariable logistic regression model (2). We did not repeat this analysis in our cohort as TARN did not comprehensively record an important mortality confounder - pre-injury antithrombotic medication status - until the end of 2019. As TBI is the predominant injury in both cohorts the result likely would be similar.  We have also added further to the “interpretation of findings “ section of discussion” to address this point made by the reviewer about the appropriateness of care received:    “Our previous study showing similar risk adjusted hospital mortality in low fallers in major trauma centres versus trauma units is limited to a short-term outcome. Longer follow up for patient reported outcomes in future major trauma in older people studies, is needed to determine whether the current less intense care pathway for older fallers with severe injuries is appropriate or represents therapeutic nihilism.” |
